# Office bladder distention with Electromotive Drug Administration (EMDA) is equivalent to distention under General Anesthesia (GA)

**DOI:** 10.1186/1471-2490-5-14

**Published:** 2005-11-22

**Authors:** Amy E Rose, Kathryn J Azevedo, Christopher K Payne

**Affiliations:** 1 Department of Urology, Stanford University Medical School, Stanford, California, USA

## Abstract

**Background:**

Bladder distention is commonly used in diagnosis and treatment of interstitial cystitis (IC). Traditionally performed in the operating room under general or spinal anesthesia (GA), it is expensive and associated with short term morbidity. Office bladder distention using electromotive drug administration (EMDA) has been suggested as an alternative that is well tolerated by patients. We report the first comparative findings of patients undergoing both office distention with EMDA and distention in the operating room (OR) with GA.

**Methods:**

This retrospective chart review identified 11 patients participating in two protocols of EMDA bladder distention who also underwent bladder distention under GA either prior to or after the EMDA procedure.

**Results:**

The median absolute difference in bladder capacity between GA and EMDA was only 25 cc; the median percent difference was 5%. Cystoscopic findings, while not prospectively compiled, appear to have been similar.

**Conclusion:**

This study represents the first comparison between distention with EMDA versus GA and confirms the technical feasibility of performing bladder distention in an office setting. The distention capacity achieved in the office was nearly identical to that in the OR and the cystoscopic findings very similar. Further investigation into the comparative morbidity, cost, and other outcome measures is warranted to define the ultimate role of EMDA bladder distention in the clinical evaluation and care of patients with IC.

## Background

Cystoscopy with bladder distention has traditionally been regarded as the diagnostic standard for interstitial cystitis (IC). Although there is considerable debate over the true value of bladder distention in the diagnosis of IC, the National Institute of Diabetes, Digestive and Kidney diseases (NIDDK) criteria require the presence of post-distention mucosal glomerulations (also referred to as "positive cystoscopy") or a bladder ulcer in order to qualify patients for clinical trials [1]. In addition, the International Continence Society terminology document limits the use of the term "interstitial cystitis" to patients "with typical cystoscopic and histological findings" [2]. Thus, while bladder distention is being used less commonly in the United States, it continues to be a central part of the diagnostic algorithm in most of the rest of the world [3]. Bladder distention can also provide symptomatic relief for some IC patients and is thus a commonly performed procedure for patients presenting with urinary frequency, urgency and bladder pain. The procedure is typically performed in the operating room (OR) under spinal or general anesthesia (GA) and is associated with moderate short term morbidity such as pain and hematuria. Moving bladder distention into an office setting could potentially eliminate the inherent risks of anesthesia, lower the cost of the procedure, and minimize the recovery period. In turn, this could make a complete evaluation including bladder distention available to a broader spectrum of symptomatic patients and make re-treatment practical for the subset of patients who respond favorably.

Our research group has focused on trying to develop office bladder distention as a realistic alternative to bladder distention under GA. An initial trial compared two different in-office anesthetic strategies: pre-operative intravesical alkalized lidocaine as described by Henry and colleagues [4] and lidocaine administered via electromotive drug administration (EMDA). We found that simple alkalinized lidocaine was completely inadequate for office bladder distention but that EMDA presented a promising technology. However, while we were able to perform a technically adequate bladder distention, some of our results [5] were quite different from prior studies published by Rosamilia and colleagues [6] and by Riedl [7]. We found that the majority of subjects (64%) anesthetized with EMDA were able to tolerate a 60 cm H_2_O distention for the full 7 minutes, and we were able to obtain a median percent increase in distention capacity over cystometric capacity of 135%. However, in contrast to prior reports that described office distention with EMDA as well tolerated, our subjects reported a median pain score at end distention of 9/10. We concluded that EMDA anesthesia was satisfactory in most cases for diagnostic in-office distention but pain was still an issue and true comparability between an EMDA distention and a distention under GA was not established.

No data exists to establish that a distention in the office with EMDA is actually equivalent to the procedure performed in the OR. We present the first report of a direct comparison between distention with EMDA versus distention with GA in a group of patients undergoing both procedures.

## Methods

Two prospective protocols have been conducted to investigate the utility of EMDA anesthesia for office bladder distention. Both were approved by the Institutional Review Board. The first examined the role of EMDA distention in the initial diagnosis of IC; two patients from this protocol later went on to have a bladder distention under GA. The second protocol examined the efficacy of EMDA distention in treating patients who had previously responded to a distention in the operating room with GA. Nine subjects have enrolled in this study. The current study population is composed of these 11 patients who have experienced bladder distention in both settings.

All research carried out on human subjects was in compliance with the Helsinki Declaration. The research protocol and consent forms were approved by the Institutional Review Board (IRB) for Stanford University Medical School and informed consent was obtained from all subjects. EMDA distention was performed as an office procedure without intravenous sedation. Most patients were given oral medications for pain, anxiety, and antibiotic prophylaxis (hydrocodone 1–2 tablets, lorazepam 1 mg, ciprofloxacin 500 mg) one hour prior to the procedure. EMDA was performed as previously described [5]. Cystoscopy and distention were performed using a 15 French flexible cystoscope. The bladder was distended at 60 cm H_2_O under direct vision with a goal of 7 minutes distention time as measured from the start of water flow and then drained under direct vision. Pressures above 60 cm H_2_O for the EMDA procedure have not been attempted as not all patients could tolerate the full 7 minute distention. Pain scores were recorded during distention using a 0–10 Likert Scale. For the seven patients with Hunner's ulcers, serum lidocaine levels were obtained.

Most of the bladder distentions under GA were performed by the senior investigator (CKP) using a standard technique. Diagnostic cystoscopy was performed with a 22 Fr rigid cystoscope after which the bladder was distended at 80 cm H_2_O for 7 minutes under direct vision then drained under direct vision. The anesthetic technique (spinal or general) was chosen by the patient and anesthesiologist. When the prior bladder distention was performed by an outside urologist the study data was obtained from the operative note.

The comparative data was collected from the most recent distention with GA and the first EMDA distention for those patients in the treatment protocol. The patients from the diagnostic protocol had only one procedure with each technique. The date of the procedure, the distention technique, pre- and post-distention findings, and bladder capacity were recorded.

## Results

Patient demographics and results are displayed in Table 1. The median age was 52 years (range 22–72); there were 6 women and 5 men. Most of the patients recruited for the treatment study had already had at least 3 previous IC therapies and many were considered end stage patients with ulcers and low bladder capacities under anesthesia. The time elapsed between the GA distention and the EMDA distention varied widely between patients with a median time of 10 months between the two procedures (range 1–91).

**Table 1:** Patient Demographics and Distention Results

**Patient**	**Age**	**Sex**	**Ethnicity**	**Hunner's Ulcer**	**General Anesthesia Capacity (cc) ** **80 cm H_2_O**	**EMDA Office Capacity (cc) ** ** 60 cm H_2_O**	**Absolute Difference Between OR and EMDA Capacity (cc)**	**% Difference**	**Pain Score During Office Distention (10)**
1	61	F	White	+	300	260	40	13	7
2	52	F	Hispanic	+	275	255	20	7	9
3	68	F	White	+	200	205	5	2	10
4	49	F	White	-	900	1105	205	19	8
5	55	M	White	-	400	350	50	12	7
6	50	F	White	-	725	725	0	0	9
7*	72	M	Asian	+	290	370	80	22	8
8*	22	M	African American	-	875	850	25	3	8
9	48	F	Indian	+	580	550	30	5	3
10	51	M	White	+	400	400	0	0	8
11	59	M	White	+	310	300	10	3	8
**Median**	**52**	-	-	-	-	-	**25**	**5**	**8**

*Office distention performed prior to operating room distention. Of 11 IC patients experiencing distention both in the OR and in the office with EMDA, the distention capacity obtained in the office at 60 cm H_2_O very closely approximates that achieved with general anesthesia at 80 cm H_2_O. In patients #3,4, and 7, the capacity achieved in the office was higher than that achieved in the OR. In patients #6 and 10, the capacity achieved was exactly the same.

The bladder capacities achieved with the two different techniques were strikingly similar; the median absolute difference was only 25 cc and the median percent difference was 5%. In no case was the EMDA capacity less than 87% of the capacity achieved with GA. In three patients, we were able to achieve a higher capacity with EMDA, and in two patients, the capacities were exactly the same.

Serum lidocaine levels were drawn from the seven patients with bladder ulcers; all were less than 1.1 μg/mL. The median pain score during the distention with EMDA was 8 on a 10 point scale (range 3–10). In most patients, pain resolved rapidly after draining the bladder at the end of distention. No patient required any parenteral medication or any additional intravesical therapy for pain. Because the GA distention data was collected retrospectively we can not make comparisons about the patient experiences during the post-operative recovery.

## Discussion

This is the first published comparison of bladder distention with EMDA versus GA. The data demonstrate that EMDA provides an equivalent degree of distention to the standard procedure performed in the operating room as essentially the same bladder capacity is achieved. These results confirm and enhance prior reports suggesting that office distention with EMDA is a viable alternative for select IC patients. Moving bladder distention into an office setting eliminates the risks of general anesthesia, almost certainly significantly reduces cost, and makes complete evaluation available to a wider spectrum of symptomatic patients. This is only a small retrospective series; a large scale prospective randomized controlled trial would be required to fully investigate all of the relevant factors including diagnostic utility, cost, morbidity, and therapeutic effect.

Although the distention capacities achieved in the office were almost identical to those achieved with GA, pain at the end of distention remains a problem. Patients were given low dose pre-emptive analgesia, but inevitably the pain became intense during the last one or two minutes of the distention. For most patients, however, the pain was transient and they were able to leave the office without assistance within an hour and resume full normal activities rapidly. Our pretreatment analgesic protocol was relatively conservative and could be increased without moving to conscious sedation. Most patients who have experienced both procedures prefer the transient pain of distention with EMDA to the pain and typically longer recovery associated with distention under GA.

There are important limitations to the current study. First, our study population is small and is not representative of the overall IC population. The patients are self selected for willingness to participate in an office protocol in order to avoid general anesthesia. This might lead one to believe there would be a selection bias toward patients with milder disease, but in fact most of the patients in this study are relatively refractory patients who failed standard therapies. Several of the patients would be considered "end stage" patients with severe ulcer disease and diminishing bladder capacity. The severity of disease in this patient population, however, emphasizes the potential value of EMDA anesthesia. For example, patient number 3 in Table 1 has multiple ulcers and a bladder capacity of only 200 cc at 80 cm H_2_O under general anesthesia, but with EMDA we were able to achieve a distention capacity of 205 cc at only 60 cm H_2_O with the patient fully awake. Second, it should also be noted that the time between the distention under GA and the distention with EMDA is quite variable among patients. Since most patients had EMDA second, if interval treatment improved the disease process that could bias the EMDA capacities to be higher. However, interval progression of disease could cause the opposite effect. Finally, showing identical bladder capacity with the two techniques does not prove equivalence in diagnostic or therapeutic utility. We could not report therapeutic results in this retrospective study. While we felt the cystoscopic findings are similar, a prospective study with a blinded third party analyzing the images would be required to prove this.

## Conclusion

This is the first reported direct comparison between office bladder distention with EMDA versus traditional distention with general anesthesia performed in the operating room. In this select group, EMDA provided an equivalent degree of distention and thus deserves further investigation to define its ultimate clinical role. Although there is a rapid recovery, pain levels during treatment remain high in our hands. A randomized trial would be required to fully explore all of the relevant factors in comparing EMDA to GA for bladder distention.

## List of abbreviations

IC: interstitial cystitis

NIDDK: National Institute of Diabetes, Digestive and Kidney diseases

OR : operating room

GA: general anesthesia

## Declaration of competing interests

The author(s) declare that they have no competing interests.

## Authors' contributions

*Amy Rose*: Made substantial contributions to conception and design, acquisition of data, analysis and interpretation of data. Was the primary person involved in drafting the manuscript and revising it critically for important intellectual content. Has given final approval of the version to be published. Involved in patient recruitment, performed the EMDA treatments, and was the primary author of the paper.

*Kathryn Azevedo*: Made substantial contributions to conception and design, acquisition of data, analysis and interpretation of data. Was involved in drafting the manuscript and revising it critically for important intellectual content. Has given final approval of the version to be published. Assisted in EMDA treatment and was in charge of all communication with the IRB and maintaining appropriate consent and documentation for the study.

*Christopher Payne*: Made substantial contributions to conception and design, acquisition of data, analysis and interpretation of data. Was involved in drafting the manuscript and revising it critically for important intellectual content. Has given final approval of the version to be published. Primary study investigator, supervised all EMDA treatments, performed OR distentions.

## Pre-publication history

The pre-publication history for this paper can be accessed here:


